# Assessment of impacted canine patterns and correlations with craniofacial discrepancy

**DOI:** 10.2340/aos.v85.45907

**Published:** 2026-05-05

**Authors:** Salma Abbas, Lale Taner, Orhan Özdiler, Erhan Özdiler

**Affiliations:** a Department of Orthodontics, Faculty of Dentistry, Cyprus Health and Social Sciences University – KSTU, Güzelyurt, Turkey; b Department of Orthodontics, Faculty of Dentistry, Gazi University, Ankara, Turkey

**Keywords:** impacted maxillary canine, panoramic radiography, cephalometric analysis, craniofacial discrepancy, skeletal morphology

## Abstract

**Objective:**

The objective of this study was to determine the prevalence, spatial patterns, and craniofacial skeletal associations of impacted maxillary canines using panoramic and cephalometric analyses in a large patient cohort.

**Materials and methods:**

This retrospective cross-sectional study evaluated 9593 panoramic radiographs of individuals aged ≥ 14 years. Cephalometric analysis was achieved on subjects aged ≥ 18 years with skeletal maturity (Cervical Vertebral Maturation Index stage ≥ 6), comparing 94 cases with maxillary impacted canines (MIC) to 100 age- and sex-matched controls. Positional measurements (sector, depth, and angulation) were compared with skeletal patterns using independent t-tests, analysis of variance, and chi-square tests (α = 0.05).

**Results:**

Analysis revealed a maxillary canine impaction prevalence of 2.02%, with a significant female predilection (54.1%) and a maxilla-to-mandible ratio of 4:1. The predominant spatial pattern was mesioangular inclination (67%) in Sector 0 (35.2%) at coronal depth (54.1%). Compared to controls, MIC cases exhibited significant maxillary skeletal deficiency (e.g., anterior nasal spine – posterior nasal spine [ANS-PNS]: 50.1 ± 5.1 mm vs. 53.1 ± 3.7 mm; *p* < 0.001) and vertical hyperdivergence (Sella-Nasion to Gonion-Gnathion angle [SN-GoGn]: 32.8° ± 6.2 vs. 29.8° ± 6.4; *p* = 0.001). Upper incisor inclination showed no intergroup difference.

**Conclusions:**

Maxillary canine impaction is strongly associated with maxillary skeletal deficiencies and vertical growth discrepancies. The common finding of mesioangular impactions in Sector 0 at coronal depth indicates a high risk for adjacent root resorption. We recommend incorporating cephalometric screening (focusing on ANS-PNS < 50 mm and SN-GoGn > 32°) into early adolescent assessments to identify at-risk patients and guide timely interceptive strategies such as maxillary expansion.

## Introduction

Maxillary canine impaction remains a clinically relevant issue in modern orthodontics and has substantial effects on occlusal function, cosmetic balance, and long-term dental health. Recent extensive meta-analyses have validated its position as the second most common dental impaction worldwide, impacting 1.5–3.2% of the population and exhibiting a consistent female predominance (ratio 1.8:1). Undiagnosed impactions provide considerable risks, including neighboring dental root resorption (28–42% prevalence) and cystic degeneration [1–3]. This vulnerability arises from the canine’s distinctive developmental trajectory: delayed onset (usually at 4–5 years), extended eruption through thick maxillary bone (exceeding 18 months), and intricate three-dimensional course [3, 4].

Although cone-beam computed tomography (CBCT) provides enhanced three-dimensional localization, traditional two-dimensional radiography, especially panoramic and lateral cephalometric imaging, continues to be the worldwide diagnostic standard in clinical practice owing to its accessibility, cost-effectiveness, and established protocols. Panoramic radiographs facilitate accurate risk stratification via established classification systems: Ericson and Lindauer sectors quantitatively assess horizontal displacement concerning the lateral incisor, Yamamoto classes delineate sagittal-vertical positions, and angulation/depth metrics consistently forecast resorption risk and treatment complexity [5, 6].

Recent studies have redefined canine impaction, viewing it not just as a dental irregularity but as an indicator of systemic craniofacial dysmorphology [7]. Advanced imaging studies have consistently correlated impaction with distinct skeletal phenotypes, including maxillary hypoplasia, transverse deficiency, and vertical hyperdivergence [8, 9]. Morphological restrictions physically restrict the osseous area for canine eruption and concurrently interfere with the biomechanical guiding processes that facilitate appropriate tooth movement [8]. However, despite these advancements, significant knowledge deficiencies remain in the literature. The statistical power of existing studies is frequently insufficient for robust phenotype identification, and this limitation is compounded by the fact that less than 20% of studies effectively incorporate panoramic localization data alongside cephalometric skeletal analysis, and the three-dimensional relationships between impaction depth/sector position and craniofacial growth patterns remain insufficiently quantified [10]. Furthermore, most studies are statistically underpowered (sample sizes < 200), limiting robust phenotype-impaction correlations. The precise relationships between specific impaction characteristics (sector, depth, angulation) and craniofacial growth patterns remain poorly quantified, hindering the development of evidence-based interceptive protocols.

Our objectives are fourfold: first, to ascertain definitive population-level prevalence and positional characteristics through panoramic screening; second, to identify unique cephalometric skeletal signatures in impacted individuals; third, to establish correlations between two-dimensional radiographic features and three-dimensional craniofacial growth patterns; and fourth, to create clinically actionable depth-stratified risk profiles for early intervention.

The present study was conceived to address the identified gaps in knowledge by testing three fundamental hypotheses derived from the contemporary understanding of maxillary canine impaction (MCI) as a dentoskeletal phenomenon. First, we hypothesized that MCI exhibits a significant association with a defined skeletal phenotype, specifically characterized by sagittal maxillary deficiency (evidenced by reduced Anterior Nasal Spine – Posterior Nasal Spine [ANS-PNS] and Condylion – A Point [Co-A] dimensions) combined with a hyperdivergent vertical facial pattern (indicated by elevated Sella-Nasion to Gonion-Gnathion angle [SN-GoGn] and Frankfort Mandibular Plane angle [FMA angles]). Second, it was postulated that specific, quantifiable features of the impaction visible on panoramic radiography – its mesiodistal sector, vertical depth, and angulation – demonstrate systematic correlations with distinct underlying craniofacial morphotypes. Third, we proposed that a systematic synthesis of panoramic localization data and cephalometric skeletal analysis could generate clinically applicable risk-stratification protocols to guide interceptive treatment.

## Materials and methods

This retrospective cross-sectional study obtained approval from the Cyprus Health and Social Sciences University Ethics Committee ID (KSTU/2024/315) on 19 April 2024 and followed the principles of the Declaration of Helsinki.

A cohort of 9593 anonymized panoramic radiographs (patients aged 14 years and older) from the institutional database (January 2020 to February 2025) was examined. Exclusion criteria: syndromic disorders, craniofacial deformities, previous orthodontic interventions, and distorted images. Cephalometric analysis was restricted to patients aged ≥ 18 years with confirmed Cervical Vertebral Maturation Index (CVM) Stage 6 – validated as the biological threshold for skeletal maturity [11]. Complete epiphyseal fusion at this stage ensures cessation of clinically significant craniofacial growth (< 0.5 mm/year mandibular change), enabling reliable evaluation of adult endpoint morphology. Chronological age alone was deemed insufficient; CVM ≥ 6 confirmation prevented misinterpretation of transitional anatomy and dynamic growth artifacts. The study cohort comprised 94 patients with ≥ 1 impacted maxillary canine and lateral cephalograms, alongside age- and sex-matched controls without impaction (*n* = 100, ± 2 years).

The demographic characteristics of the cephalometric groups were as follows. The maxillary impacted canines (MIC) group included 94 subjects (mean age 22.1 ± 3.8 years; 58 females, 36 males). The control group consisted of 100 subjects (mean age 21.7 ± 3.5 years; 60 females, 40 males). No significant differences in age or sex distribution were found, confirming successful matching.

### Sample size justification

A post-hoc power analysis was performed using G*Power (v.3.1.9.7). Using the primary outcome of maxillary length (ANS-PNS), a mean difference of 2.5 mm and a pooled standard deviation of 4.5 mm (derived from a comparable study) yielded an effect size (d) of 0.56. With the final sample sizes (*n* = 94 cases, *n* = 100 controls) and an alpha of 0.05, the achieved statistical power was 0.92 (92%), confirming the sample was adequate to detect a significant difference.

Images were obtained via the Kavo Orthopantomograph OP 3D Type PCX_1 (Kavo Dental GmbH, Germany): Panoramic radiographs: 68–85 kVp, 10–15 mA, 14–18 seconds exposure time. Cephalograms: 75–90 kVp, 15 mA, 150 cm source-to-midsagittal distance. Monthly calibration guaranteed uniform magnification (cephalograms: 8%; panoramics: 25%).

Impaction was diagnosed based on the absence of complete eruption beyond the age of 15, confirmed by radiographic evidence of obstruction along the eruption path. Two calibrated orthodontists independently assessed the radiographs, achieving high inter-examiner reliability (Cohen’s κ > 0.85), ensuring diagnostic consistency and accuracy.

Radiographic analysis of ectopically erupting maxillary canines was performed.

The vertical position of the impacted canine was categorized relative to the adjacent lateral incisor root using perpendicular bisectors dividing the root’s long axis into thirds:

**Apical:** Located toward the root apex (lowest third)

**Middle:** Positioned at the mid-root level (central third)

**Figure 1:**
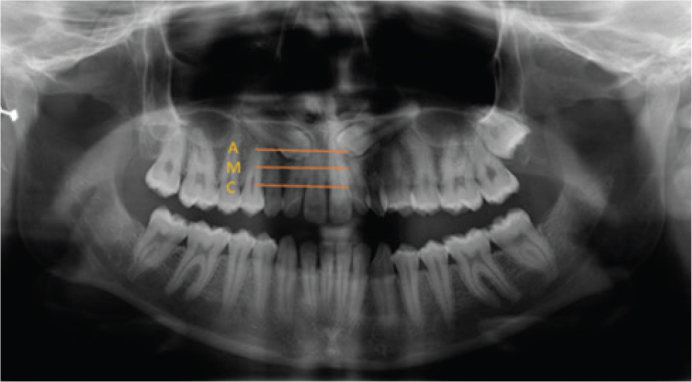
The depth of the impacted canine. A: apical, M: middle, C: coronal. Classification of impaction depth was performed according to the criteria established by Ericson and Kurol [6].

**Coronal:** Situated toward the cementoenamel junction (uppermost third Figure 1)

**Figure 2:**
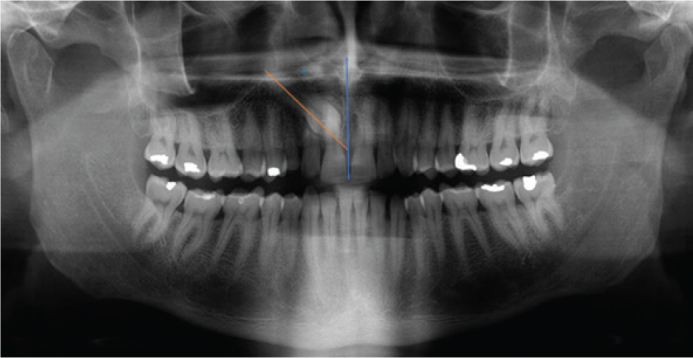
The angle of the impacted canine related to the midline. Angulation relative to the midline was categorized based on the method described by Ericson and Kurol [6].

**Angulation:** The angle formed between the long axis of the canine and the occlusal plane: vertical (75–90°), mesioangular (> 90°), distoangular (< 75°), or horizontal (< 30° Figure 2).

**Figure 3:**
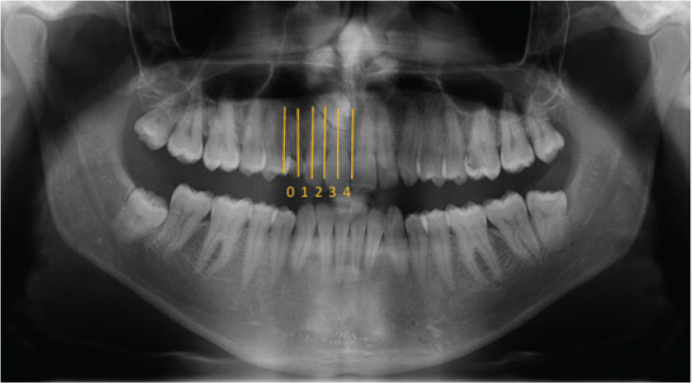
The sectors of the impacted canine mesio-distally. Mesiodistal sector classification followed the method described by Ericson and Kurol [6].

The mesiodistal position of the impacted canine on panoramic images was classified using the sector method (Sectors 0–10). As the majority of impactions occurred within Sectors 0–4, cases were grouped into three categories for comparative cephalometric analysis: Group 0 (Sector 0), Group 1–2 (Sectors 1–2), and Group 3–4 (Sectors 3–4). Comprehensive cephalometric analyses were then performed for each group to investigate potential associations between the location of impaction within the dental arch and distinct skeletal or dental patterns (Figure 3).

**Cephalometric Examination.** Tracings using VistaDent™ software (v.6.1) by a blinded evaluator (ICC > 0.92):

- **Cranial base:**

**N-S** (Nasion-Sella Line).

- **Maxillary measurements:**

**ANS-PNS**, (Anterior Nasal Spine – Posterior Nasal Spine): Maxillary length

**Co-A**, (Condylion – A Point): Effective maxillary length

- **Vertical parameters:**

**SN-GoGn** (Sella-Nasion to Gonion-Gnathion angle): Mandibular plane angle.

**FMA angle**, (Frankfort Mandibular Plane angle).

**Jarabak ratio.** (Sella-Gonion / Nasion-Menton × 100): Facial height ratio.

- **Sagittal relationships:**

**SNA** (Sella-Nasion-A Point angle): Maxillary sagittal position.

- **Dental inclinations:**

**U1-NA** (Upper incisor to Nasion-A point): Measured in both millimeters/degrees.

**Figure 4:**
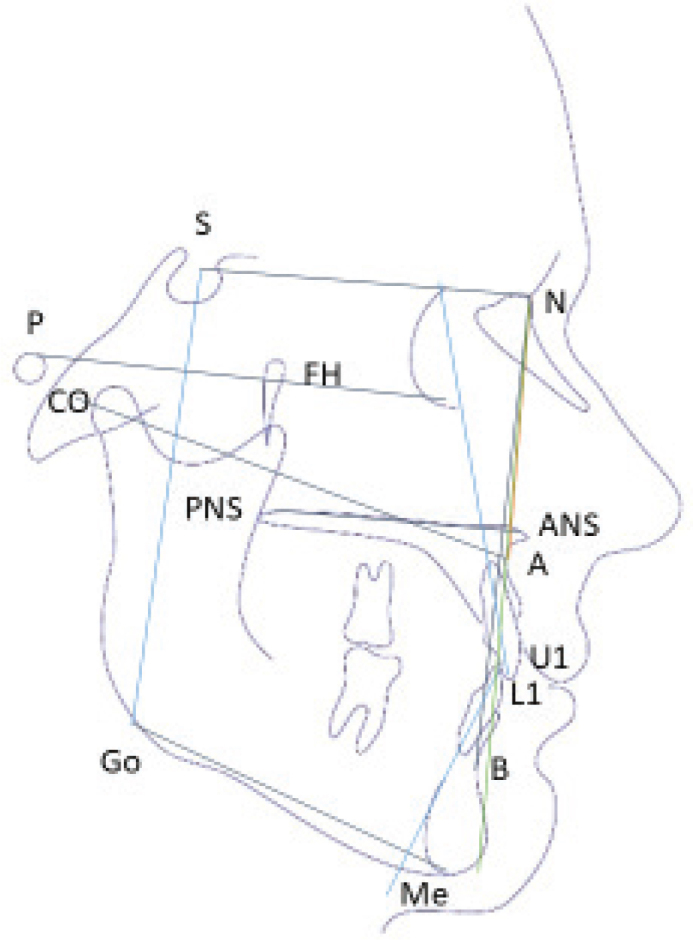
The cephalometric analysis lines and angles used.

**L1-NB** (Lower incisor to Nasion-B point): Measured in both millimeters/degrees (Figure 4).

### Reliability assessment

Inter-examiner reliability for panoramic classifications was assessed by comparing the independent initial assessments of the two orthodontists for 30 randomly selected radiographs. Intra-examiner reliability was evaluated by having the primary examiner re-evaluate the same 30 radiographs after a 4-week interval, blinded to the initial measurements. For cephalometric measurements, intra-examiner reliability was similarly tested on a subset of 30 tracings after a 4-week washout period.

### Statistical analysis

Data was analyzed in IBM SPSS v.27 (Armonk, NY: IBM Corp.). Continuous variables: mean ± SD (range); categorical: frequencies (%). Normality was assessed via Kolmogorov-Smirnov and Shapiro-Wilk tests. Comparisons: Independent *t*-tests (two groups) Analysis of Variance (ANOVA) with Bonferroni post hoc (≥ 3 groups) Chi-square/Fisher’s exact tests (categorical) Statistical significance: two-tailed *p* < 0.05.

Intergroup comparisons employed independent *t*-tests (with Cohen’s *d* for effect size) for two groups, one-way ANOVA (with Bonferroni post hoc tests and partial eta-squared) for three or more groups, and chi-square or Fisher’s exact tests for categorical variables. Non‑parametric alternatives (Mann‑Whitney U, Kruskal‑Wallis H) were reserved should assumptions be violated.

Reliability was assessed using Cohen’s Kappa for categorical classifications (sector, depth, angulation) and intraclass correlation coefficients (ICC) for continuous cephalometric measurements. Intra‑examiner reliability was excellent (ICC > 0.89; Dahlberg’s error < 1.0° or 1.0 mm). A two‑tailed *p*‑value < 0.05 denoted statistical significance.

## Results

Among 9593 screened individuals, 2.27% (*n* = 218; 95% CI: 1.9–2.59%) exhibited canine impaction, with maxillary impactions predominating (1.75%, *n* = 194) versus mandibular (0.48%, *n* = 46). Females accounted for 54.1% (*n* = 118) of cases versus males (45.9%, *n* = 100). Unilateral impactions (73.4% overall) showed right-side predominance (41.3%, *n* = 90), followed by left-side (32.1%, *n* = 70), while bilateral cases represented 26.6% (*n* = 58) (Table 1).

**Table 1:** Prevalence and demographic distribution of canine impactions.

Parameter	Category	No.	%
**Overall prevalence**	-	218/9593	2.27
**Arch**	Maxillary	194	2.02
	Mandibular	46	0.48
**Sex**	Female	118	54.1
	Male	100	45.9
**Distribution**	Unilateral	160	73.4
	- Right	90	41.3
	- Left	70	32.1
	Bilateral	58	26.6

Among 233 impacted maxillary canines evaluated, the most common angulation was mesioangular (67.0%), followed by vertical 26.6%, horizontal 3.9%, and disto-angular as the rarest at 2.6%. Sector distribution showed the highest frequency in Sector 0 (35.2%), followed by Sectors 3, 1, 4, and Sector 2. Regarding depth, the majority of impactions were positioned at the coronal third of adjacent roots (54.1%), while the middle and apical thirds represented 24.5 and 21.5%, respectively (Table 2).

**Table 2:** Positional characteristics of impacted maxillary canines (*n* = 233).

Parameter	Category	Number	%
**Angulation**	Mesioangular	156	67.0
	Vertical	62	26.6
	Horizontal	9	3.9
	Distoangular	6	2.6
**Sector**	0	82	35.2
	1	34	14.6
	2	24	10.3
	3	52	22.3
	4	32	13.7
	Other*	9	3.9
**Depth**	Coronal	126	54.1
	Middle	57	24.5
	Apical	50	21.5

* Sectors –3, –1, 5, and 7 were grouped as ‘Other’ due to low frequency (*n* = 9).

Cephalometric findings showed that patients with impacted maxillary canines had significantly shorter anterior cranial base (N-S: 64.3 ± 4.2 vs. 67.7 ± 3.2 mm, *p* < 0.001), palatal maxillary length (ANS–PNS: 50.1 ± 5.1 vs. 53.1 ± 3.7 mm, *p* < 0.001), and diminished basal maxillary length (Co-A: 78.5 ± 6.1 vs. 82.3 ± 4.8 mm, *p* < 0.001), along with a reduced SNA angle (80.9° ± 4.4 vs. 82.3° ± 3.5, *p* = 0.014). Vertically, the impaction group exhibited a more hyperdivergent pattern, with significantly increased SN-GoGn (32.8° ± 6.2 vs. 29.8° ± 6.4, *p* = 0.001), FMA (24.3° ± 5.9 vs. 21.6° ± 6.4, *p* = 0.004), and a decreased Jarabak ratio (65.4% ± 5.8 vs. 68.1% ± 5.0, *p* = 0.002). No significant differences were observed in incisor inclination (U1-NA) (Table 3).

**Table 3:** Consolidated cephalometric findings in maxillary impacted canine cases.

Cephalometric variable	MIC cases (*n* = 94)	Controls (*n* = 100)	*P*
**Sagittal skeletal**			
N-S (mm)	64.3 ± 4.2	67.7 ± 3.2	**< 0.001**
ANS-PNS (mm)	50.1 ± 5.1	53.1 ± 3.7	**< 0.001**
Co-A (mm)	78.5 ± 6.1	82.3 ± 4.8	**< 0.001**
SNA (°)	80.9 ± 4.4	82.3 ± 3.5	**0.014**
**Vertical skeletal**			
SN-GoGn (°)	32.8 ± 6.2	29.8 ± 6.4	**0.001**
FMA (°)	24.3 ± 5.9	21.6 ± 6.4	**0.004**
Jarabak ratio (%)	65.4 ± 5.8	68.1 ± 5.0	**0.002**
**Dental**			
U1-NA (mm)	5.5 ± 2.6	5.6 ± 2.4	**0.962**
U1-NA (°)	21.5 ± 8.7	23.4 ± 8.3	**0.121**

MIC: maxillary impacted canine; N-S: Nasion-Sella Line; ANS-PNS: Anterior Nasal Spine – Posterior Nasal Spine; Co-A: Condylion – A Point; SN-GoGn: Sella-Nasion to Gonion-Gnathion angle; SNA: Sella-Nasion – A Point angle; FMA angle: Frankfort Mandibular Plane angle; Jarabak ratio: Sella-Gonion/Nasion-Menton × 100: Facial height ratio; U1-NA: Upper incisor to Nasion-A point.

### Subgroup analyses within the impaction cohort

Further investigation into the MIC cohort revealed specific skeletal associations. Female patients demonstrated significantly reduced maxillary dimensions (ANS-PNS, Co-A) and cranial base length (N-S) compared to their male counterparts (*p* < 0.005). Furthermore, regardless of gender, cases with bilateral impactions showed more severe sagittal skeletal deficiencies, with significantly shorter N-S and ANS-PNS lengths, compared to unilateral cases (*p* < 0.03).

The specific characteristics of the impaction were also linked to skeletal patterns. Vertically impacted canines were strongly associated with a hyperdivergent skeletal phenotype, marked by significantly greater SN-GoGn and FMA angles and a lower Jarabak ratio (*p* < 0.02). In contrast, the mesiodistal sector location on panoramic radiographs showed no significant correlation with any cephalometric parameters.

A key finding emerged from the analysis of impaction depth. The middle-depth impaction group was characterized by a significantly more horizontal growth pattern, with a lower FMA and a higher Jarabak ratio, compared to both the coronal and apical depth groups. This group also displayed the lowest SN-GoGn angle, a finding that approached statistical significance (*p* = 0.050). The key subgroup cephalometric comparisons are summarized in Table 4

**Table 4:** Summary of significant subgroup cephalometric comparisons within the MIC cohort.

Subgroup comparison	Key finding	*P*
**Sex (Female vs. Male)**	Females had significantly shorter N-S, ANS-PNS, and Co-A.	< 0.005
**Laterality (Bilateral vs. Unilateral)**	Bilateral cases had significantly shorter N-S and ANS-PNS.	< 0.03
**Angulation (Vertical vs. Angulated)**	Vertical impactions had significantly higher SN-GoGn, higher FMA, and lower Jarabak Ratio.	< 0.02
**Depth (Middle vs. Apical/Coronal)**	Middle-depth impactions had significantly lower FMA and higher Jarabak Ratio.	< 0.03

MIC: maxillary impacted canine; N-S: Nasion-Sella Line; ANS-PNS: Anterior Nasal Spine – Posterior Nasal Spine; Co-A: Condylion – A Point; SN-GoGn: Sella-Nasion to Gonion-Gnathion angle; FMA angle: Frankfort Mandibular Plane angle.

All measurements are presented as mean ± standard deviation. *p* < 0.05 is considered statistically significant.

**Abbreviations:** FMA, Frankfort Mandibular Plane Angle; SN-GoGn, Sella-Nasion to Gonion-Gnathion Angle; Jarabak Ratio, (Sella-Gonion / Nasion-Menton) × 100.

## Discussion

This work directly challenges these constraints by conducting an extensive dual-modality radiography investigation of 9593 patients, constituting the largest cohort analyzing panoramic localization and cephalometric phenotypes yet collected to examine skeletal-impaction relationships.

This large-scale investigation elucidates the multifactorial etiology of MCI, confirming its strong association with specific craniofacial morphological patterns. The findings substantiate the central hypothesis that MCI is not merely a localized dental anomaly but a frequent clinical manifestation of an underlying skeletal form, principally characterized by maxillary deficiency and a hyperdivergent vertical growth pattern.

### Epidemiological and demographic foundations

The prevalence of MCI in our cohort (2.02%) corroborates the established global range of 1.0–3.0%, affirming its status as a common clinical challenge [12]. The pronounced 4:1 maxilla-to-mandible ratio underscores the unique biomechanical vulnerability of the maxillary canine, attributable to its extended and tortuous eruption path through dense anterior maxillary bone – a susceptibility now well-documented through 3D analyses of its gubernacular canal [13]. The observed female predilection (54.1%) aligns with persistent epidemiological trends and is likely rooted in sexually dimorphic craniofacial development. This predisposition may arise from an interplay of earlier canine maturation and relatively reduced maxillary arch dimensions in females, creating a potential mismatch between dental eruption timing and skeletal space availability [14, 15].

### Positional characteristics and local risk

Analysis of the 233 impacted canines revealed consistent positional trends with direct clinical implications. Mesioangular inclination was predominant (67%), a finding typically attributed to premature contact with the root of the lateral incisor, which deflects the normal eruptive vector. In the mesiodistal plane, impactions most frequently occurred in Sector 0 (35.2%), followed by Sectors 3 and 1, indicating a tendency for displacement near the midline and lateral incisor. This upper anterior localization is critical, as it correlates with a significantly elevated risk of severe root resorption to the adjacent teeth [16]. Vertically, coronal-third placements were most common (54.1%), positioning the impacted canine crown at the vulnerable cervical third of the lateral incisor root – a zone particularly prone to resorptive damage under sustained pressure [17].

### The defining skeletal phenotype in MCI

The most salient contribution of this study is the definitive characterization of the skeletal phenotype associated with MCI. Cephalometric analysis confirmed that affected individuals exhibit a dual pattern of maxillary deficiency – evidenced by significantly reduced ANS-PNS length, Co-A distance, and SNA angle – and vertical hyperdivergence, indicated by steeper SN-GoGn and FMA angles. This phenotype creates a hostile environment for eruption: transverse and sagittal deficiency physically constricts the available osseous pathway, while vertical excess likely elongates the eruption course and may diminish functional occlusal guidance. This aligns with contemporary evidence repositioning MCI as a sign of underlying skeletal form rather than an isolated event [10, 18]. The absence of significant differences in incisor inclination or sagittal occlusion classification between groups further redirects etiology from dental compensation to the foundational skeletal framework.

### Differential skeletal correlates of impaction phenotypes

Subgroup analyses revealed nuanced skeletal correlations that aid in risk stratification. The more pronounced maxillary deficiency observed in female patients provides a plausible skeletal explanation for their higher impaction prevalence, echoing findings from 3D CBCT studies [19]. Furthermore, cases of bilateral impaction were linked to more severe sagittal maxillary and cranial base deficits than unilateral cases, suggesting a stronger systemic or potentially genetic influence in these patients, consistent with associations reported for genes like MSX1 and PAX9 [20].

Importantly, the skeletal pattern also correlated with the clinical presentation of the impaction itself. Vertically oriented impactions showed a strong association with hyperdivergent morphology. A particularly insightful finding was the link between impaction depth and vertical skeletal pattern: middle-depth impactions were associated with a more hypodivergent, brachyfacial pattern (higher Jarabak ratio, lower FMA), while coronal and apical impactions correlated with hyperdivergence. This suggests the vertical facial type may influence the level at which the canine’s eruption is arrested. Conversely, the lack of significant cephalometric differences across mesiodistal sectors implies that the bucco-palatal position (sector) is governed more by local dental factors (e.g., lateral incisor root morphology) than by the overarching skeletal pattern.

Our findings provide immediately applicable protocols for clinicians to proactively identify high-risk patients during routine panoramic screenings, particularly females exhibiting maxillary hypoplasia (ANS-PNS < 50 mm) alongside vertical hyperdivergence (SN-GoGn > 37°), facilitating timely morphology-guided intervention before irreversible complications arise [21]. This approach radically alters the diagnostic paradigm from reactive localisation to proactive skeletal risk evaluation.

### Clinical recommendations

Based on these findings, we advocate for a proactive screening protocol that integrates skeletal assessment into routine orthodontic evaluation. For adolescent patients, an SN-GoGn angle > 32° should serve as a general cephalometric risk indicator, with a threshold of > 37° warranting early interceptive measures in high-risk subgroups. Specifically, females aged 10–12 years with ANS-PNS < 50 mm and SN-GoGn > 37° represent a priority group for targeted intervention, including maxillary expansion to address sagittal and transverse deficiency. While CBCT remains essential for complex case management, our study confirms that 2D panoramic and cephalometric imaging provide adequate initial risk stratification, promoting radiation-conscious clinical practice.

### Limitations

1. Two‑dimensional radiography lacks CBCT accuracy for assessing root proximity; future 3D imaging studies are needed.
2. Single‑center design may limit generalizability; multi‑ center studies should confirm findings.
3. Cross‑sectional design prevents causal inference; longitudinal research must validate cephalometric predictors and genetic factors.

## Conclusions

1. Maxillary canine impaction (MCI) as a manifestation of specific craniofacial morphology, primarily characterized by maxillary skeletal deficiency (reduced ANS-PNS) and vertical hyperdivergence (elevated SN-GoGn).
2. The predominant clinical presentation – mesioangular inclination in Sector 0 at coronal depth – signals a high risk for adjacent root resorption.
3. Risk stratification reveals distinct patient profiles:
  - Females exhibit more severe maxillary skeletal deficits.
  - Bilateral impaction cases demonstrate greater sagittal deficiencies compared to unilateral cases.
  - Impaction depth correlates with vertical facial pattern: middle-depth impactions are associated with a more horizontal growth pattern, while coronal and apical impactions align with hyperdivergence.
4. These findings advocate for a paradigm shift from reactive management to proactive, morphology-based screening in orthodontic practice.
5. We recommend integrating cephalometric analysis (ANS-PNS < 50 mm; SN-GoGn > 32°–37°) with panoramic evaluation for at-risk adolescents to enable timely interception, reserving advanced 3D imaging for complex cases.

## Data Availability

The datasets used and analyzed during the current study are available from the corresponding author at saaalma8s@gmail.com upon reasonable request.
